# Lung ultrasound in the emergency department - a valuable tool in the management of patients presenting with respiratory symptoms during the SARS-CoV-2 pandemic

**DOI:** 10.1186/s12873-020-00389-w

**Published:** 2020-12-07

**Authors:** Bonaventura Schmid, Doreen Feuerstein, Corinna N. Lang, Katrin Fink, Rebecca Steger, Marina Rieder, Daniel Duerschmied, Hans-Jörg Busch, Domagoj Damjanovic

**Affiliations:** 1 Department of Emergency Medicine, University Hospital of Freiburg, Faculty of Medicine, University of Freiburg, Freiburg, Germany; 2 Heart Center Freiburg University, Department of Cardiology and Angiology I, Faculty of Medicine, University of Freiburg, Freiburg, Germany; 3 Department of Medicine III (Interdisciplinary Medical Intensive Care), Medical Center, Faculty of Medicine, University of Freiburg, Freiburg, Germany; 4 Heart Center Freiburg University, Department of Cardiovascular Surgery, Faculty of Medicine, University of Freiburg, Freiburg, Germany

**Keywords:** Lung ultrasound, COVID-19, Triage, Emergency department

## Abstract

**Background:**

Typical lung ultrasound (LUS) findings in patients with a COVID-19 infection were reported early on. During the global SARS-CoV-2 pandemic, LUS was propagated as a useful instrument in triage and monitoring. We evaluated LUS as a rapid diagnostic triage tool for the management of patients with suspected COVID-19 in the emergency department (ED).

**Methods:**

The study retrospectively enrolled patients with suspected COVID-19, who were admitted from 1st April to 25th of April 2020 to the ED of a tertiary care center in Germany. During clinical work-up, patients underwent LUS and polymerase chain reaction (PCR) testing for SARS-CoV-2. The recorded ultrasound findings were analyzed and judged regarding typical signs of viral pneumonia, blinded for clinical information of the patients. The results were compared with PCR test and chest computed tomography (CT).

**Results:**

2236 patients were treated in the ED during the study period. 203 were tested for SARS-CoV-2 using PCR, 135 (66.5%) underwent LUS and 39 (28.9%) of the patients were examined by chest CT scan. 39 (28.9%) of the 135 patients were tested positive for SARS-CoV-2 with PCR.

In 52 (38.5%) COVID-19 was suspected from the finding of the LUS, resulting in a sensitivity of 76.9% and a specificity of 77.1% compared with PCR results. The negative predictive value reached 89.2%. The findings of the LUS had - compared to a positive chest CT scan for COVID-19 - a sensitivity of 70.6% and a specificity of 72.7%.

**Conclusions:**

LUS is a rapid and useful triage tool in the work-up of patients with suspected COVID-19 infection during a pandemic scenario. Still, the results of the LUS depend on the physician’s experience and skills.

## Background

Detection of pneumothorax, acute decompensated heart failure, bacterial and viral pneumonia as well as pulmonary embolism with lung ultrasound (LUS) has extensively been evaluated, and has been implemented in point-of care work-up in emergency departments (ED) as well as intensive care units (ICU) [1, 2].

During the SARS-CoV-2 pandemic, several publications postulated the use of LUS as a helpful diagnostic tool in the management of patients with a suspected infection with SARS-CoV-2 [3–5]. In case reports and case series, typical imaging patterns of viral pneumonia suggesting COVID-19 were postulated [6, 7].

In the face of the current pandemic, EDs were confronted with a large cohort of patients with suspected COVID-19. The gold standard for diagnosing SARS-CoV-2-infection is a polymerase chain reaction (PCR)-test of a nasopharyngeal or oropharyngeal swab, with the disadvantage of a minimum testing time of one to several hours, depending on laboratory capacities. PCR is not always easily available in every institution, and has the risk of false-negative results [8]. In contrast, point-of-care LUS is broadly available and can be performed on a bedside manner at any time by the attending physician without additional contacts and patient transportation.

Patients in the ED who are waiting for test results lead to crowding, which carries an additional risk of disseminating the infection. In this situation, LUS may provide a rapid and feasible triage tool in suspected COVID-19. LUS also offers a tool to immediately identify patients who are at risk of respiratory failure and should be referred to an ICU due to a more pronounced pulmonary affection [9]. Additionally, repeated LUS can be used to monitor patients during the hospital stay, and help determine the appropriate level of care, escalate or de-escalate respiratory support and/or indicate proning, e.g. LUS also offers the great advantage to identify alternative causes for respiratory symptoms, e.g. pneumothorax or acute heart failure [9].

Radiologic imaging is a routine diagnostic technique for suspected pneumonia. But in chest X-rays a great amount (up to 27%) of pneumonias (otherwise detectable in a computed tomography (CT)) remain undetected [10]. Therefore, chest CT scans were declared as the preferred imaging technique for COVID-19 [11]. COVID-19 typical patterns in chest CT are spotty bilateral ground glass opacities in mild cases. In moderate to severe cases, the density increases gradually, with small subpleural consolidations mainly located in the caudal and dorsal lung areas [11]. CT provides concise imaging of the lung parenchyma, but goes along with radiation, costs, limited availability in some areas and includes extra staff and transports. An imaging technology without the aforementioned risks but with the advantage of bedside (“point of care”) use is LUS.

Because of the frequent pleural involvement and preferably peripheral distribution of pathology, lesions typical of viral pneumonia, as described in COVID-19, can be detected with sonography. Therefore, LUS was evaluated as a triage tool in patients with a potential SARS-CoV-2 infection in all-comer environments. To our knowledge, this study evaluated the role of LUS for the diagnosis in the ED in a lager cohort for the first time.

## Methods

In the ED of the University Hospital of Freiburg (Germany), LUS was established as part of the routine work-up of patients with a suspected infection with SARS-CoV-2 during the preparations for the evolving pandemic. Patients with respiratory symptoms were treated in a separated part of the ED (the ‘respiratory area’). Patients were allocated according to symptoms for a respiratory infection, which were fever, cough, dyspnea and chest pain. Additionally, patients with contact to a person with a SARS-CoV-2 infection and patients who returned from countries, which were on the list of risk areas of the Robert Koch Institut (RKI), were also allocated to the separated part of the ED.

All attending physicians (doctors in training for emergency medicine, consultants for emergency medicine) in the ED had experience in point-of-care ultrasound as a standard diagnostic tool. They were additionally trained following a standardized protocol in order to perform LUS in patients with suspected COVID-19 and recognize typical patterns (Table 1).

**Table 1:** LUS signs that would suspect COVID-19 disease and LUS signs that are unlikely for COVID-19

COVID-19 suspected	COVID-19 unlikely
bilateral patchy distribution of one of the following:	**unilateral appearance of one of the following:**
• pleural line irregularity OR	• **singular pleural line irregularities OR**
• ≥ 3 B-Lines per intercostal space OR	• **≥ 3 B-Lines per intercostal space in a homogenous distribution (e.g. basal lung parts) OR**
• small subpleural consolidation (< 1.5 cm) OR	• **larger subpleural consolidations (> 1.5 cm) OR**
• no or small pleural effusion	• **large pleural effusion OR**
unilateral appearance of two or more of the criteria above	

For documentation, a 12-zone model was adopted with two anterior, two lateral and two posterior zones, separated by the parasternal line, anterior axillary line, posterior axillary line and the middle of the lung, respectively. The trainings were completed by the end of March for all ED team members.

### Patient enrollment and data retrieval

From 1st of April 2020 to 25th of April 2020, we retrospectively enrolled all admitted patients who were treated in the respiratory pathway in the ED due to respiratory symptoms and initially suspected infection. Clinical work-up, PCR-testing for SARS-CoV-2, LUS and - if carried out - chest CT scans were analyzed. The charts were reviewed for data on symptoms, previous conditions, laboratory findings and outcome after 4 weeks (hospitalization, intensive care treatment and death). Every suspected case of a SARS-CoV-2 infection was tested using PCR. Regarding the CT scans a radiologist approved the final diagnosis following a state-of-the-art work-up.

### Data processing

Three experts (D.F., B.S., D.D.) with experience in LUS re-evaluated the descriptive LUS findings and ultrasound images, which were accomplished by the ED physicians beforehand. The experts were blinded to all clinical information about the patient, especially for the SARS-CoV-2-PCR test result, other clinical findings, past medical history and previous conditions, as well as other imaging findings of the CT. They judged, according to typical patterns in LUS (Table 1) for COVID 19. If their finding met the criteria for COVID-19, they diagnosed the infection only based on the LUS. If judgment differed between the three, cases were re-read together and agreement on one diagnosis was achieved.

The final diagnoses by LUS were correlated with the PCR results. As a subgroup analysis, the LUS results were compared to patients’ chest CT scans which had to be done during the first 48 h after ED admission.

### Statistical analysis

Variables following Gaussian distribution were compared using student’s t-test, non-normally distributed continuous values by using Mann-Whitney-U test. Categorical variables were assessed by chi-square test or Fisher’s exact test as appropriate. All calculations were performed with SPSS, version 26 (IBM, NYC, USA).

## Results

During the study period, out of 2236 ED admissions, 203 patients with respiratory symptoms were allocated to a “respiratory pathway” and consecutively tested for SARS-CoV-2, or were transferred from primary care doctors with persistent clinical impairment due to already confirmed SARS-CoV-2 infection (*n* = 24). We excluded 68 (33.5%) patients who did not have documented LUS in the record or did probably not undergo LUS.

**Fig. 1:**
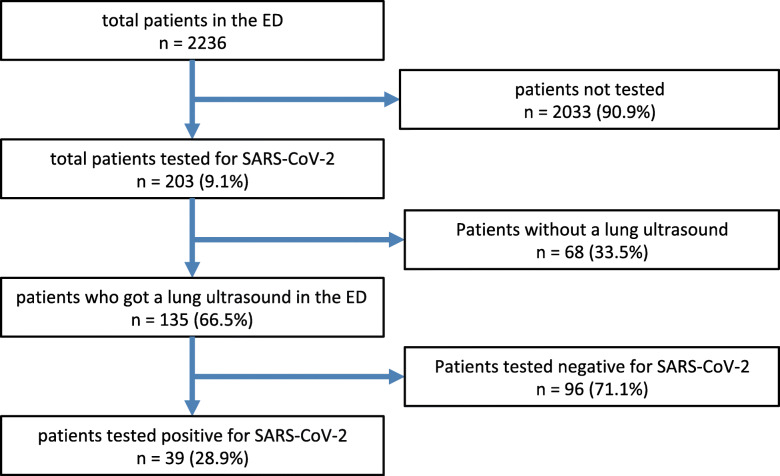
Patients in the ED during the study period in absolute numbers, percentage refer to the number above. Every suspected case of a SARS-CoV-2 infection was tested using PCR

135 (66.5%) patients underwent LUS and were included in the study. Out of this group, 96 patients (71.1%) were tested negative for SARS-CoV-2 by PCR, 39 patients (28.9%) were tested positive, or were known positive (Fig. 1).

### Baseline characteristics

The medium age of the analyzed group was 61 ± 18 (18–95) years, 73 (54.1%) were male, 98 (72.6%) had to be admitted to the hospital. Significantly more patients with SARS-CoV-2 were admitted to a ward (*p* = 0.046), of whom 16 (11.9%) were treated in an intensive care unit (Table 2). 12 (8.9%) died during the hospital stay within 4 weeks since admission. No differences were detected in the vital signs on admission. Symptoms on admission significantly differed between the two groups. Cough as well as limb pain were more frequent in the SARS-CoV-2 positive group, whereas fever was more often detected in the group negative for SARS-CoV-2. Significantly more patients with an oncological disease or a pre-existing chronic pulmonary disease were observed in the group tested negative for SARS-CoV-2 (Table 2).

**Table 2:** Patient characteristics on hospital admission. *p*-values refer to the comparison between the SARS-CoV-2 negative and the SARS-CoV-2 positive patients. ^a^ presented as median ± standard deviation (range), ^b^ number of patients (percentage based on the number of all patients), ^c^ based on Mann-Whitney-U test for nonparametric variables, ^d^ based on chi-square test / Fisher’s exact test as appropriate for categorical variables

	SARS-CoV-2 positive (*n* = 39)	SARS-CoV-2 negative (*n* = 96)	*p*-value
patient characteristics			
age [years]	61 ± 16 (25–84) ^a^	60 ± 18 (18–95) ^a^	0.774 ^c^
sex [male]	22 (56.4%) ^b^	51 (53.1%) ^b^	0.728 ^d^
BMI [kg/m^2^]	26.3 ± 6.6 (15.8–52.5) ^a^	26.3 ± 6.2 (16.4–46.9) ^a^	0.722 ^c^
vital sign on admission			
MAD [mmHg]	101,4 ± 14 (71–145) ^a^	98.3 ± 16.6 (58–133) ^a^	0.361 ^c^
heart rate [/min]	**84.8 ± 16.6 (42–113)** ^**a**^	**94.8 ± 22 (42–148)** ^**a**^	**0.034** ^**c**^
temperature [°C]	37.7 ± 0.9 (36.0–39.4) ^a^	37.5 ± 1.0 (35.0–40.1) ^a^	0.101 ^c^
symptoms at admission			
fever	30 (23.1%) ^b^	44 (45.8%) ^b^	0.001 ^d^
cough	**26 (66.7%)** ^**b**^	**35 (36.5%)** ^**b**^	**0.003** ^**d**^
dyspnea	25 (64.1%) ^b^	57 (59.4%) ^b^	0.610 ^d^
chest pain	6 (15.4%) ^b^	19 (19.8%) ^b^	0.550 ^d^
limb pain	**8 (20.5%)** ^**b**^	**4 (4.2%)** ^**b**^	**0.002** ^**d**^
gastrointestinal symptoms	11 (28.2%) ^b^	22 (22.9%) ^b^	0.517 ^d^
medical history			
hypertension	19 (48.7%) ^b^	43 (44.8%) ^b^	0.678 ^d^
chronic heart failure	3 (7.7%) ^b^	13 (13.5%) ^b^	0.341 ^d^
diabetes	5 (12.8%) ^b^	17 (17.7%) ^b^	0.611 ^d^
chronic kidney injury	**2 (5.1%)** ^**b**^	**19 (19.8%)** ^**b**^	**0.037** ^**d**^
oncological disease	**7 (17.9%)** ^**b**^	**35 (36.5%)** ^**b**^	**0.035** ^**d**^
lung disease	**4 (10.3%)** ^**b**^	**33 (34.4%)** ^**b**^	**0.005** ^**d**^
smoking (ongoing/ previous)	3 (7.7%) ^b^	22 (22.9%) ^b^	0.050 ^d^
laboratory results at admission			
leukocyte count (tsd/μl) (*n* = 135)	**6.96 ± 4.71 (1.05–26.80)**^**a**^	**10.57 ± 7.13 (0.44–49.71)** ^**a**^	**< 0.001** ^**c**^
platelet count (tsd/μl) (*n* = 135)	**189.46 ± 88.04 (10.00–490.00)** ^**a**^	**245.05 ± 124.92 (8.00–699.00)** ^**a**^	**0.010** ^**c**^
hemoglobin g/dl (n = 135)	**13.03 ± 1.98 (8.80–17.10)** ^**a**^	**11.91 ± 2.49 (4.38–16.40)** ^**a**^	**0.003** ^**c**^
creatinine mg/dl (n = 135)	1.09 ± 0.66 (0.37–4.18) ^a^	1.31 ± 1.31 (0.49–10.50) ^a^	0.627 ^c^
C-reaktive protein mg/l (n = 135)	71.1 ± 77.1 (3.0–291.9) ^a^	69.6 ± 82.9 (3.0–374.3) ^a^	0.571 ^c^
procalcitonin ng/ml (*n* = 129)	0.16 ± 0.21 (0.05–0.95) ^a^	1.67 ± 8.55 (0.05–81.60) ^a^	0.223 ^c^
lactat dehydrogenase U/l (n = 135)	**369 ± 216 (141–1108)** ^**a**^	**285 ± 155 (137–1163)** ^**a**^	**0.006** ^**c**^
proBNP (ng/l) (*n* = 127)	1296 ± 666 (50–18,995) ^a^	3004 ± 832 (50–5733) ^a^	0.085 ^c^
d-dimer (mg/l) (*n* = 98)	3.53 ± 1.55 (0.19–35.20)^a^	2.17 ± 0.45 (0.19–21.57) ^a^	0.945 ^c^
inpatient care			
hospital admission	**33 (84.6%)** ^**b**^	**65 (67.7%)** ^**b**^	**0.046** ^**d**^
ICU admission	5 (12.8%) ^b^	11 (11.5%) ^b^	0.777 ^d^
death	6 (15.4%) ^b^	6 (6.3%) ^b^	0.104 ^d^

Leukocytes and thrombocytes were significantly lower in the SARS-CoV-2 group. Lactate dehydrogenase was significantly higher in the group that was tested positive. On the other hand, hemoglobin was significantly lower in the group tested negative for SARS-CoV-2. The C-reactive protein and the procalcitonin did not differ significantly between groups.

In the evaluation of the LUS findings 52 (38.5%) patients exhibited patterns consistent with COVID-19. 83 (61.5%) findings were considered not typical of COVID-19. The decision of the experts was made by consensus in 93 (68.9%) cases, in 42 (31.1%) cases disagreements were resolved by discussion.

The sensitivity of LUS indicating the diagnosis of COVID-19 was 76.9% and the specificity was 77.1% according to a positive swab. The positive predictive value was calculated as 57.7% and the negative predictive value as 89.2%. The comparison of LUS to chest CT readings resulted in a sensitivity of 65.0% and a specificity of 72.7% (Table 3).

**Table 3:** Crosstable with the relation between suspected infection with LUS and PCR-test on SARS-CoV-2 and secondly the relation between suspected infection with LUS and findings in the CT

Sign of COVID-19 in the lung ultrasound			
	LUS with suspected COVID-19 disease	LUS without suspicion for COVID-19 disease	
SARS-CoV-2 PCR-test			
positive	**30**	**9**	39
negative	**22**	**74**	96
	52	83	135
Sign of COVID 19 in the Chest CT			
yes	**13**	**7**	20
no	**3**	**8**	11
	16	15	31

Those patients with LUS findings suspicious for COVID-19 but with a negative PCR (*n* = 22) were further analyzed. In 16 cases a complete chart-review provided the following alternative diagnoses: pulmonary malignancy (*n* = 5), other infections (*n* = 4), decompensated heart failure (*n* = 3), pulmonary embolism (n = 3) and pulmonary emphysema with atelectasis (*n* = 1). When excluding these 16 cases from the analyses, specificity of LUS increased to 92.5% and the positive predictive value increased to 83.3%.

## Discussion

LUS is an established point-of-care tool for the evaluation of patients in the ED. [12] In the last years, LUS was evaluated for several diagnoses and established in the clinical work-up. In diagnosing pneumothorax, LUS showed an excellent specificity and sensitivity [13, 14]. For the detection of pneumonia LUS can achieve a specificity of 75–90% and a sensitivity of 85–95% [15]. LUS is superior to standard chest X-ray in detecting or ruling-out pulmonary edema [16]. Standard use of LUS in the evaluation of pulmonary embolism is not recommended, but it can be used as a decision tool [17].

During the evolving SARS-CoV-2 pandemic, LUS was recommended as a diagnostic tool for patients with COVID-19 [3]. At the moment, only case series are published, no high-yielding study evaluated the role of LUS in diagnosing COVID-19 so far [18]. The sonographic signs of COVID-19 are similar to other viral pneumonias [16, 19]. There is no evidence for a pathognomonic LUS COVID-19-sign, but findings in LUS like bilateral b-lines, pleural irregularity and/or subpleural consolidations lead to an increased suspicion of COVID-19 disease.

In the ED of the University hospital of Freiburg, LUS was part of the initial work-up of patients with respiratory symptoms and suspected SARS-CoV-2 during the pandemic. The indication for testing for SARS-CoV-2 infection was also based on respiratory symptoms and suspected infections.

The implemented protocol in the ED focused on feasibility and a rapid execution in a point-of-care manner for evaluation of a COVID-19 diagnosis. The physician who treated the patients also performed the LUS. The findings were documented in the records. CT of the chest was only performed in severe cases with suspected superinfection or other causes for respiratory impairment (e.g. pulmonary embolism). A strict indication procedure for a chest CT scan limited the case number of additional chest CT scans, and was part of the strategy to reduce contact and spread of infections within the hospital [20].

In most publications concerning LUS, only highly skilled doctors performed the examination. In this study, treating physicians had different experience levels in LUS but nevertheless they were able to perform exams with a sufficient quality to identify suspected COVID-19 infections. No patients with pre-existing pulmonary diseases or a more likely differential diagnosis were excluded because it was the intention to show the ED reality with simultaneously occurring diseases during the pandemic.

In this setting, LUS achieved a moderate sensitivity (76.9%) and specificity (77.1%), and a high negative predictive value (89.2%). In symptomatic patients, LUS markedly decreases suspicion of COVID-19 pneumonia as the underlying cause of respiratory symptoms. But cannot exclude SARS-CoV-2 infection in asymptomatic patients, or if there is no relevant involvement of the lung in a positive patient [9]. This limits the LUS to the use as a screening tool for patients with leading respiratory symptoms. This aspect can also be a limitation for the CT as a screening tool.

The expert team was blinded to the clinical circumstances and findings. Thus, misinterpretations of LUS findings from alternative causes like acute heart failure or pulmonary malignancy could have been interpreted as suspected infection. Hereby, we wanted to emphasize the image-based judgement. In real life, LUS has to be interpreted in the clinical context by the physician [9]. Nevertheless, sensitivity and specificity are acceptable and the data shows that the LUS can be used as a screening tool with a high negative predictive value.

Patients with false-positive LUS were closer evaluated, showing that many of them would have probably been judged differently if the clinical circumstances and the precondition would have been known. The exclusion of these patients led to an even higher specificity and a positive predictive value in a sub-analysis.

CT of the chest is the gold standard for the detection of pulmonary impairment in patients with COVID-19 [21]. The results of LUS also have an acceptable sensitivity and specificity when compared to the CT results. Using a CT scan in every patient is cost- and resource intense, would expose many patients unnecessarily to radiation and could lead to further spread of the infection [20]. Additionally, CT is not easily available for the treating physicians in many ambulatory settings.

Furthermore, as no radiation is necessary, LUS can easily be repeated later in the course of the disease and the treatment. Changes in conditions can be easily monitored, and deterioration of the patient can be quickly evaluated. Thus, it is also suitable in pregnancy and for children.

In the view of the pandemic, LUS turned out to be broadly available, cost-effective and provides rapid results for further clinical screening, triage and treatment. For primary care doctors, LUS may be a useful tool for the treatment and evaluation of ambulatory patients or patients in nursing homes, when test results of SARS-CoV-2 swabs are pending.

### Limitations

The total sample size of the study as well as the SARS-CoV-2 positive subset of patients are small. Additionally, results are dependent on documentation and interpretation, which might be compromised by the physicians’ experience. Missing documentation led to the exclusion of 68 patients treated during the study period in the ‘respiratory area’ of the ED.

## Conclusions

LUS is a rapid and feasible tool in the ED and in the ambulatory setting with broad availability. It has an adequate sensitivity and specificity in detecting signs of COVID-19 pneumonia with a high negative predictive value. Most physicians with general experience in ultrasound only need a short training in LUS including the typical patterns of COVID-19 to achieve correct results.

LUS may be used as a screening tool in the ambulatory setting in patients with a suspected infection, especially if PCR testing is not easily available or pending.

We strongly recommend that LUS should be further evaluated as a screening and monitoring tool in larger prospective clinical trials. As in all user-dependent tools, the results of the LUS depend on the experience of the provider.

## Data Availability

The datasets during and/or analyzed during the current study available from the corresponding author on reasonable request.

## Abbreviations

COVID-19: Coronavirus disease 19
CT: Computed tomography
ED: Emergency department
ICU: Intensive care unit
LUS: Lung ultrasound
PCR: Polymerase chain reaction
SARS-CoV2: Severe acute respiratory syndrome coronavirus 2
